# Fabrication and Characterization of Electrospun PCL-MgO-Keratin-Based Composite Nanofibers for Biomedical Applications

**DOI:** 10.3390/ma8074080

**Published:** 2015-07-07

**Authors:** Maame A. D. Boakye, Nava P. Rijal, Udhab Adhikari, Narayan Bhattarai

**Affiliations:** 1 Department of Chemical, Biological, and Bioengineering, North Carolina A&T State University, Greensboro, NC 27411, USA; E-Mails: maboakye@aggies.ncat.edu (M.A.D.B.); nprijal@aggies.ncat.edu (N.P.R.); 2 Department of Mechanical Engineering, North Carolina A&T State University, Greensboro, NC 27411, USA; E-Mail: uadhikar@aggies.ncat.edu; 3 NSF-ERC for Revolutionizing Metallic Biomaterials, North Carolina A&T State University, Greensboro, NC 27411, USA

**Keywords:** poly(ε-caprolactone), keratin, magnesium oxide, electrospinning, nanofiber, biomedical applications

## Abstract

Polymeric nanofibers are of great interest in biomedical applications, such as tissue engineering, drug delivery and wound healing, due to their ability to mimic and restore the function of natural extracellular matrix (ECM) found in tissues. Electrospinning has been heavily used to fabricate nanofibers because of its reliability and effectiveness. In our research, we fabricated poly(ε-caprolactone)-(PCL), magnesium oxide-(MgO) and keratin (K)-based composite nanofibers by electrospinning a blend solution of PCL, MgO and/or K. The electrospun nanofibers were analyzed by scanning electron microscopy (SEM), Fourier transform infrared spectroscopy (FTIR), mechanical tensile testing and inductively-coupled plasma optical emission spectroscopy (ICP-OES). Nanofibers with diameters in the range of 0.2–2.2 µm were produced by using different ratios of PCL/MgO and PCL-K/MgO. These fibers showed a uniform morphology with suitable mechanical properties; ultimate tensile strength up to 3 MPa and Young’s modulus 10 MPa. The structural integrity of nanofiber mats was retained in aqueous and phosphate buffer saline (PBS) medium. This study provides a new composite material with structural and material properties suitable for potential application in tissue engineering.

## 1. Introduction

Polymeric hybrid nanofibers are gaining popularity among biomaterial researchers and industries because of their outstanding functional properties [1,2]. These hybrid nanofibers exhibit excellent mechanical and biological properties, which make them very attractive for various biomedical applications, such as tissue engineering scaffolds, wound dressings devices, drug delivery materials, medical implants, biosensors and filtration devices [1]. Nanofibrous structures give rise to a high surface area-to-volume ratio similar to that of proteoglycans (glycosaminoglycan) and fibrous proteins, which comprise the ECM of living tissue [3,4]. Several methods have been used to fabricate nanofibers, such as drawing, self-assembly and phase separation. However, in most cases, nanofibers are typically created using the electrospinning technique, which is cost-effective and reliable [5]. This process is one of the well-established techniques to fabricate a 3D porous nanofiber-based matrix that can be easily scaled up from laboratory to commercial production. A high voltage is applied to a continuous dripping of polymeric solution, which makes the polymer surface uniformly charged, creating electrostatic repulsion forces. When the surface tension overcomes the electrostatic force, a jet is ejected as a nanofiber from the polymer droplet and is collected in the collector.

The choice of polymer material for electrospinning is guided by the desirable properties, such as biocompatibility, limited toxicity, biodegradability and sufficient mechanical/structural integrity in the corresponding fibers for various biomedical applications [6]. Several natural and synthetic polymers have been used to generate nanofibers suitable for broad applications in biomedical engineering. The synthetic polymers include polylactic acid (PLA), polyglycolic acid (PGA)-based linear aliphatic polyesters and their copolymer, poly(ε-caprolactone) (PCL), *etc*., and have been widely used [7,8]. Natural polymers, including collagen, gelatin, hyaluronan, silk, chitosan, alginate, keratin, fibrinogen and elastin, are the most commonly-used natural polymers in tissue engineering [7,9,10]. Many of these natural polymers have cell-binding sites and biomolecular signatures that can mimic natural tissue [11]. Synthetic materials usually have good mechanical properties compared to the natural polymers. Even though there are some advantages of using synthetic materials, they have limited cell affinity, primarily due to their hydrophobicity and lack of surface cell recognition sites [7,12,13]. Natural polymers when used alone do not produce nanofibers with sufficient strength. Therefore, natural polymers are blended into synthetic polymers in which the favorable biological functionality is contributed by natural polymers, whereas the mechanical stiffness is provided by their synthetic counterparts. This type of hybrid system is expected to significantly improve material properties, while providing a stable, nurturing environment for a broad array of biomedical applications [14].

In this study, we fabricated and characterized PCL-based composite nanofibers. Electrospun nanofibers were prepared from the solutions of PCL/magnesium oxide (PCL/MgO) and poly(ε-caprolactone)-keratin/magnesium oxide (PCL-K/MgO). PCL is popular in the area of biomaterials and tissue engineering [15] due to its biodegradability, biocompatibility and superior mechanical properties [16,17]. Keratin, found largely in hair and fingernails [18], has been used as the natural polymer. Keratin has intrinsic biological activity and cell binding motifs that can support cellular attachment [19]. Keratins are the main subgroup of intermediate filament proteins and are the most abundant proteins in epithelial cells [20] that contribute to the mechanical integrity of the cell and also function as regulatory protein. Keratin has also been used in several applications, such as in wound healing, drug delivery and tissue engineering. Additionally, there has been an increasing interest in magnesium and its alloys as potential biodegradable implant materials, in biomedical applications, such as for cardiovascular and orthopedic devices [21]. Magnesium possesses excellent biocompatibility, biodegrades into soluble Mg ions (Mg^2+^), which are non-toxic by-products, and has proven use as an excellent nutrient for human metabolism [22,23]. Soluble magnesium plays an important role in various cellular processes, such as cellular respiration, protein synthesis, membrane integrity, ATPase function and oxidative phosphorylation [24,25,26]. MgO is an inorganic metal oxide and is a source of magnesium supplement that is biodegradable and provides soluble Mg^2+^ ions [27]. In this study, MgO was introduced in the electrospun nanofiber, and various physical, chemical and mechanical properties were analyzed. Nanofiber morphology was analyzed by using SEM; chemical composition and surface chemistry was determined by FTIR; and the mechanical properties of nanofibers were evaluated for tensile loading. Additionally, a release study was carried out at different time points in order to quantify the amount of magnesium released from the PCL-based composite nanofibers using inductively-coupled plasma optical emission spectroscopy (ICP-OES).

## 2. Materials and Method

### 2.1. Materials

Human hair was obtained from a local barbershop in Greensboro, NC. Peracetic acid solution and Trizma^®^ base (Product Number T1503) were purchased from Sigma-Aldrich (St. Louis, MO, USA). Hydrochloric acid (A144C-212 Lot 093601) (HCl) was purchased from Fisher Scientific (Pittsburgh, PA, USA). PCL (Mn70–90 kDa) and MgO (nanopowder, <50-nm particle size) were obtained from Sigma-Aldrich (St. Louis, MO, USA). 2,2,2-Trifluoroethanol (99+%) (TFE) was obtained from Alfa Aesar (Ward Hill, MA, USA). A 21-gauge, stainless steel dispensing needle, 1.5 inches long (product number: 75165A757) and with a 1/8 inch inner diameter, fluorinated ethylene propylene tubing and Luer lock syringe needle fittings were obtained from McMaster-Carr (Atlanta, GA, USA). Five-milliliter Luer lock syringes (Catalog Number 14-829-45) were obtained from Fisher Scientific (Pittsburgh, PA, USA).

### 2.2. Keratin Extraction

The keratin extraction process was adapted from a previous method [6]. Human hair was washed with warm water and soap and rinsed off thoroughly with deionized (DI) water. Then, the hair was treated with peracetic acid solution (PAS) 2% (w/v) for 12 h at room temperature. Afterwards, the hair was separated from the PAS using a 500-µm sieve and then rinsed thoroughly with DI water to remove residual acids. PAS was used to break the disulfide bonds that are prevalent in keratin. Free proteins were extracted in 100 mM Tris base solution (TBS) for one hour, followed by a second extraction in DI water for another hour. These extractions were carried out in a Dubnoff Metabolic Shaking Incubator (Model 2876, Marietta, OH, USA) that was set to 37 °C and 65 rpm, and retained by passage through a 500-µm sieve. Extracts were neutralized to a pH of 7 using diluted HCL (1 mM), concentrated using a rotary evaporator (Heizbad, Germany), and centrifuged (VWR Clinical 200, Germany) at 3000 rpm for 10 min in order to remove particles. Extracts were dialyzed against DI water using a cellulose membrane with a 12-kDa–14-kDa molecular weight cutoff (Fisher Scientific, Pittsburgh, PA, USA). After extracts were purified for 24 h by dialysis, they were lyophilized to form a keratin powder.

### 2.3. PCL/MgO Solution Preparation

PCL was dissolved in TFE at a concentration of 10% (w/v) overnight to obtain a homogeneous mixture. Afterwards, PCL/MgO solutions were created by mixing PCL with MgO in ratios of 1:0, 1:0.05, 1:0.10, 1:0.20, 1:0.50, 1:1, 1:1.5, 1:2 and 1:3 respectively. Each solution was prepared separately by mixing in a vortex for 20 min.

### 2.4. PCL-K/MgO Solution Preparation

Lyophilized keratin powder was dissolved in DI water at a concentration of 10% (w/v). PCL was dissolved in TFE at a concentration of 10% (w/v). PCL-K solution was then created by first mixing PCL with keratin at a ratio of 90:10. This was followed by mixing PCL-K with MgO at a ratio of 1:1. The final solution mixture (*i.e.*, PCL-K/MgO) was vortexed for 20 min.

### 2.5. Electrospinning of PCL/MgO and PCL-K/MgO Nanofibers

A previously prepared solution of PCL/MgO and PCL-K/MgO was individually fed into the syringe of 10 mL and then placed into a syringe pump (Model 78-01001, Fisher Scientific, Pittsburgh, PA, USA). The syringe pump was set to a flow rate of 1–2 mL/h for the PCL/MgO and PCL-K/MgO solutions. The positive lead from the high voltage power supply (Model CZE100PN30, Spellman High Voltage Electronics Corporation, Hauppauge, NY, USA) was fixed to a 21-gauge needle, and an 11-kV voltage was applied, which charged the polymer solution. Aluminum foil was wrapped around the collector for collecting the fiber. The tip to collector distance was maintained at 10 cm. The fibers formed were deposited onto a rotating grounded collector. Nanofiber samples were collected for 2–6 h.

### 2.6. Scanning Electron Microscopy

The electrospun nanofibers were sputter coated with gold by using a Polaron SEM Coating System for 1 min and 30 s at 15 mA. Then, these nanofibers were imaged using the SEM (Hitachi SU8000, Tokyo, Japan). The samples were observed at an accelerating voltage of 10 kV and a 5-µA current. The diameter of these electrospun fibers was determined by using Image-Pro Plus 6.0 software.

### 2.7. Characterization of Electrospun Nanofibers

To characterize the chemical bonding between PCL, MgO and keratin, FTIR spectra were obtained at 64 scans using a Varian 680i FTIR (Agilent Technologies, Santa Clara, CA, USA). The nanofiber membrane was placed in a sample compartment, and the system was purged with dry air before testing. Spectrum analysis was performed using standard Microcal Origin Software (Northampton, MA, USA).

### 2.8. Mechanical Tensile Test

Mechanical properties of the nanofibers were evaluated using a Shimadzu machine (North America Analytical and Measuring Instruments AGS-X series, Columbia, MD, USA). Trapezium Lite X software was used to collect data. Load and displacement values were collected every 500 milliseconds with a 50 N load cell at a displacement rate of 10 mm/min.

Mechanical characterization of electrospun nanofiber is extremely sensitive, as it involves measurement of very small changes in load required for deformation. These nanofibers are so delicate that any direct touch of the mat surface during sample preparation and testing can damage the fibers; hence, sufficient care must be taken. In order to avoid any such damage during handling and to maintain uniformity in loading conditions, a paper template of (38 mm × 25 mm) with an opening of (6 mm × 12 mm) was prepared as described in the literature [28]. The template consisted of two halves, top and bottom, and held the sample with the aid of double-sided adhesive tape. Fiber samples were center aligned and placed within a gauge length (Lg) of a 6-mm window cut from a paper template. The axis of the specimen was maintained parallel with the load axis precisely to prevent specimen bending and premature failure. Five samples for each ratio of nanofiber were cut to a width of 5 mm and a length of 16 mm. The thickness of each sample was measured using a digital micrometer. Fiber samples were strained to breakage, and the ultimate tensile strength and Young’s modulus were derived from stress-strain curves.

### 2.9. Degradation and Release Study of Mg

Nanofibers of PCL/MgO and PCL-K/MgO were dried under reduced pressure at room temperature. These samples were incubated in 40 mL of 1X Phosphate Buffer Saline (PBS) solution for 1 week at 37 °C in a conical tube. At various time intervals, 10 mL of solution were extracted, and the medium in the tube was replenished with 10 mL of fresh 1X PBS. The concentration of Mg released in these solutions was determined using ICP-OES against standards made from High Purity Standards solutions (Catalog Number CCV-1, High Purity Standards, Charleston, SC, USA) and 2.0% nitric acid (Catalog Number CLBK-HNO3-250, SPEX CertiPrep, Metuchen, NJ, USA). These High Purity Standards were multi-element standards that contained Mg. Samples for ICP analysis were prepared with 1 mL of concentrated nitric acid (15.8 M) added to each solution. The data in ppm obtained from ICP analysis were used in determining the percent of Mg released in each solution.

Dried nanofibrous membranes were cut into squares (~30 mm × 30 mm), sterilized with 70% alcohol (10 min incubation) and washed thoroughly with DI water. Membrane stability was then tested by incubating samples in 15 mL PBS (pH 7.5 at 37 °C). The buffer was replaced every 3 days. Nanofibers after incubation for a requisite time were removed from the PBS solution, rinsed with DI water and lyophilized. For *in vitro* degradation testing, these samples were observed for morphological changes under an SEM. SEM experimental parameters were the same as discussed earlier in the SEM section.

### 2.10. Statistical Analysis

Statistical analysis was performed using a one-way analysis of variance (ANOVA). *p*-values less than 0.05 were considered statistically significant, and the Tukey test method was conducted for pairwise comparisons. SPSS Statistics 17.0 software was used to conduct the statistical analysis.

## 3. Results

### 3.1. Keratin Extraction

With the use of a peracetic acid solution, the keratin extraction process occurred through oxidation reactions. The disulfide bonds in keratin were cleaved by PAS and yielded keratin powder in a form called keratose [18]. This keratin powder was soft and had a brown color with a yield of 0.7%–2%.

### 3.2. Morphology of Composite Nanofibers

**Figure 1:**
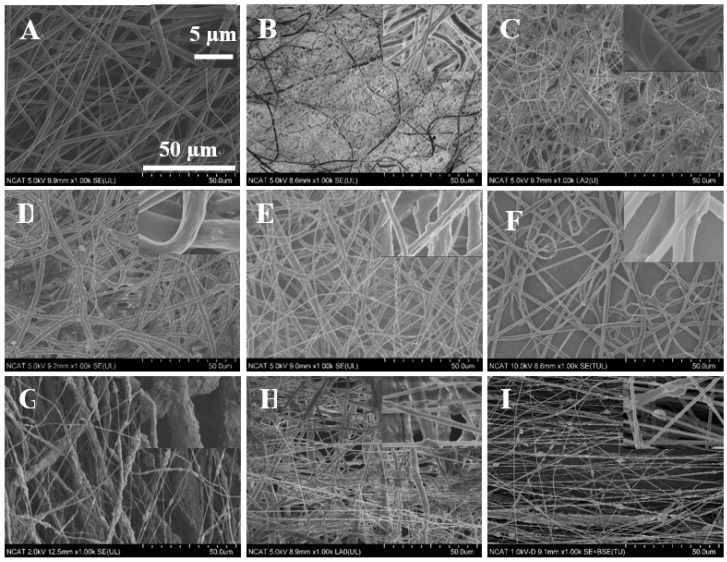
SEM images of electrospun nanofibers of poly(ε-caprolactone) (PCL)/MgO with ratios of (**A**) 1:0, (**B**) 1:0.05, (**C**) 1:0.10, (**D**) 1:0.20, (**E**) 1:0.50, (**F**) 1:1, (**G**) 1:1.5, (**H**) 1:2; and PCL-K/MgO (**I**) 1:1. The insets show higher magnification images of each corresponding SEM images. The scale bar in the inset represent 5 µm for the insets; scale units for the lower magnification are on the figures (*i.e.*, 50 µm).

The surface morphology of the various ratios of PCL/MgO nanofibers and PCL-K/MgO are shown in Figure 1. Each of the precursor solutions were able to successfully produce nanofibers by electrospinning. Figure 1A shows the structure of pure PCL nanofibers, while the remaining Figure 1B–F represents the changes in the morphology of nanofibers with increasing MgO concentration. The diameter for the PCL fiber without any MgO varied from 0.4 µm to 1.1 µm with an average value around 0.6 µm. Nanofibers at a lower MgO concentration with a PCL/MgO weight ratio less than one were free of beads, but for nanofibers with a higher MgO concentration, traces of bead formation are noticed.

**Figure 2:**
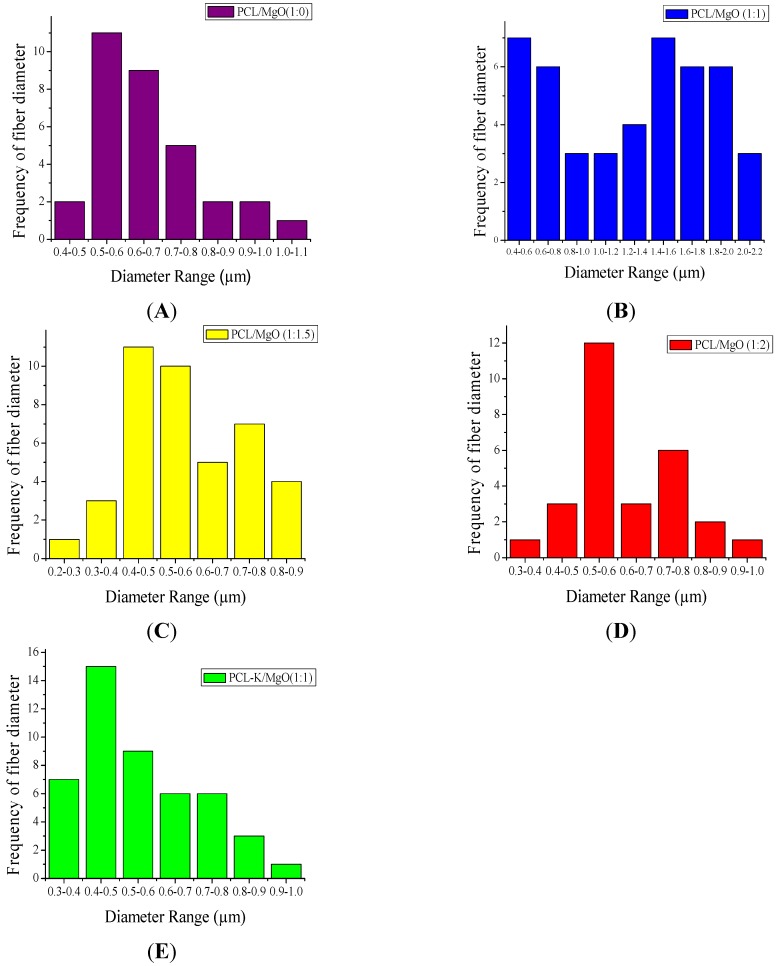
Size distribution of nanofibers: fiber diameters from SEM images of Figure 1A (**A**), Figure 1F (**B**), Figure 1G (**C**), Figure 1H (**D**), Figure 1I (**E**) were measured by using Image-Pro Plus 6.0 software.

Specifically, the frequency distribution of nanofiber diameter of PCL/MgO ratios of 1:1, 1:1.5 and 1:2, as well as the PCL-K/MgO ratio of 1:1 were further analyzed in this study. The PCL/MgO (1:1) nanofiber diameter was distributed in the range of 0.4 µm–2.2 µm; however, for the nanofibers with precursor solution of PCL/MgO (1:1.5) and PCL/MgO (1:2), the diameter distribution was in the narrower region of 0.2 µm–0.9 µm, and 0.3µm–1.1 µm, respectively, as shown in Figure 2. The PCL-K/MgO (1:1) nanofiber diameter distribution was from 0.3 µm to 0.9 µm. All of the ratios of precursor solution had the highest frequency of nanofibers distribution from 0.4 µm to 0.6 µm.

### 3.3. FTIR Analysis of the Nanofibers

**Figure 3:**
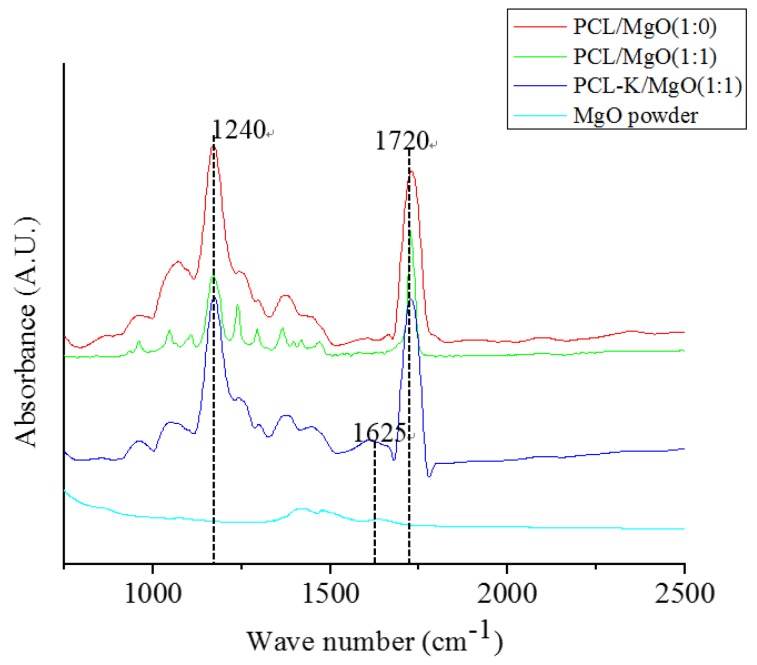
Comparative FTIR spectra of electrospun PCL/MgO and PCL-keratin/MgO nanofibers along with as-received MgO powder.

Figure 3 shows the FTIR spectra of the electrospun PCL/MgO and PCL-K/MgO nanofibers. The characteristic features of PCL and keratin in the nanofibers were observed in the spectra. PCL shows a strong absorption band at 1720 cm^−1^ corresponding to the carbonyl groups, C–O stretching at 1050 cm^−1^, and C–O–C stretching at 1240 cm^−1^ [6]. Keratin proteins give rise to amide I (1600–1700 cm^−1^) [29]. The spectra for the PCL-K/MgO (1:1) nanofiber shows an absorption band at 1625 cm^−1^, which is not seen in other compositions.

### 3.4. Mechanical Tensile Test

**Figure 4:**
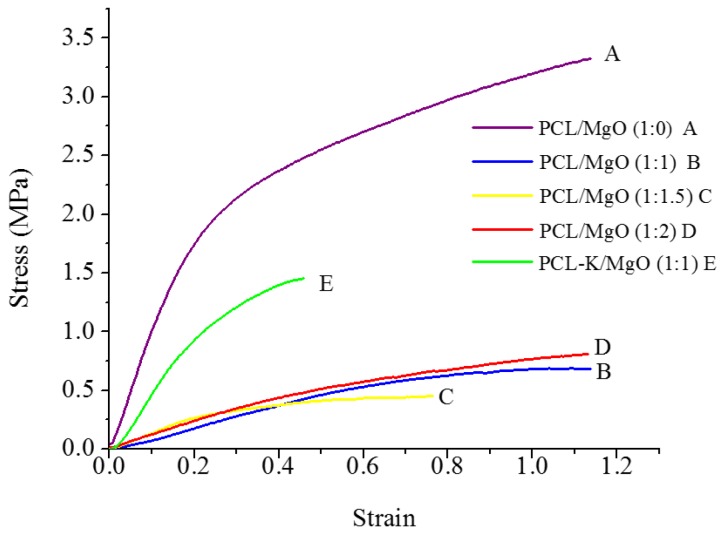
Representative stress-strain curves of PCL/MgO and PCL-K/MgO nanofibers, *n* = 5.

Tensile testing was performed on a nanofibrous membrane of PCL/MgO ratios of 1:0, 1:1, 1:1.5 and 1:2, as well as a PCL-K/MgO ratio of 1:1. Figure 4 shows the stress *versus* strain curves for the fiber samples tested. The Young’s modulus was determined using Hooke’s law from the slope of the linear portion of the stress-strain curve. The ultimate tensile strength represented the highest stress that a nanofiber sample could bear without breaking.

**Figure 5:**
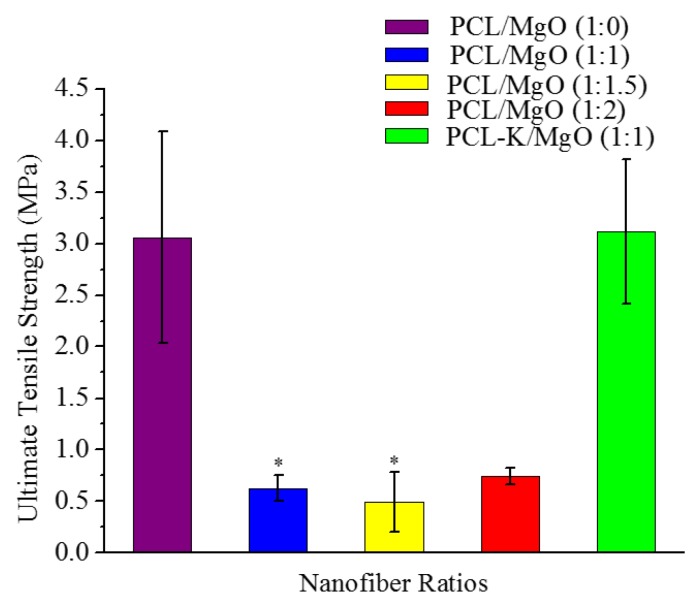
Ultimate tensile strength of PCL/MgO and PCL-K/MgO nanofibers, *n* = 5. Asterisks denote statistical significance (*p* < 0.05) compared to the PCL/MgO (1:0) nanofibers.

**Figure 6:**
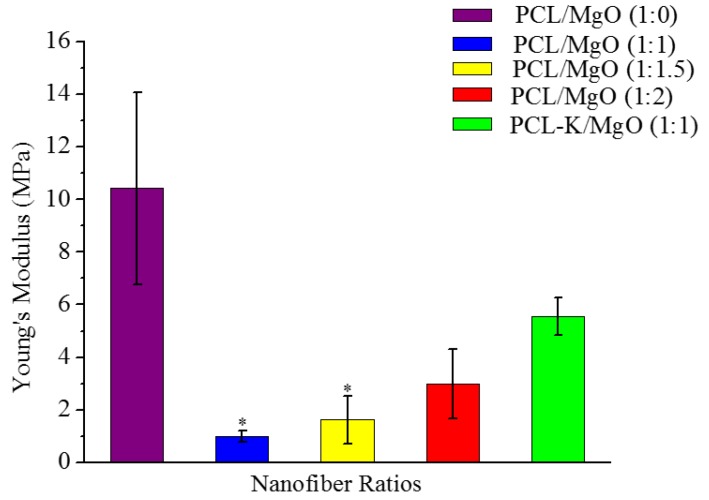
Young’s modulus of PCL/MgO and PCL-K/MgO nanofibers, *n* = 5. Asterisks denote statistical significance (*p* < 0.05) compared to the PCL/MgO (1:0) nanofibers.

Figure 5 and Figure 6 show the ultimate tensile strength and Young’s modulus obtained from stress-strain curves. There was a significant difference when comparing the ultimate tensile strength for pure PCL nanofibers with those with PCL/MgO at the *p* = 0.05 level. The strength of the nanofiber decreased after the introduction of MgO into the solution. The ultimate tensile strength for PCL/MgO ratios of 1:2, 1:1, 1:1.5 and 1:0 was found to be 0.8, 0.6, 0.5 and 3.1 MPa, respectively. The strength for the nanofiber with PCL-K/MgO in the ratio 1:1 was observed to be 3.2 MPa. Here, the addition of keratin in PCL/MgO increased the ultimate tensile strength slightly higher (3.3%) than that of pure PCL. Similarly, there was also a significant difference when comparing the Young’s modulus for PCL/MgO ratios of 1:1 and 1:1.5 to 1:0, and the comparison of Young’s modulus for the remainder of the nanofiber ratios was not significant. The Young modulus for PCL/MgO ratios of 1:2, 1:1, 1:1.5 and 1:0 was found to be 3.5, 1, 2 and 10.5 MPa, respectively. Higher values of Young modulus were observed either for pure PCL or PCL/MgO with keratin (5 MPa) when compared to PCL/MgO nanofibers without any keratin. The ultimate strength and Young modulus of PCL/MgO electrospun nanofiber was significantly improved by polyblending with the natural polymer, keratin.

### 3.5. Degradation and Release Study of Mg

**Figure 7:**
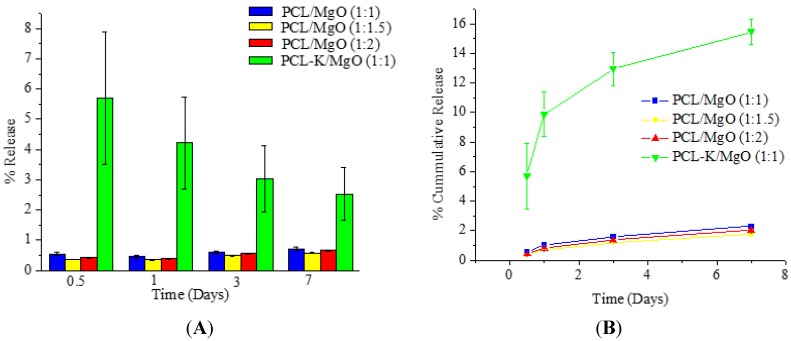
Release profile of Mg by using ICP-OES, *n* = 3. (**A**) Percentage release of each nanofiber composition over different time intervals. (**B**) Cumulative release of each nanofiber composition over different time intervals. PCL-K/MgO nanofiber samples released Mg significantly more over time compared to all other PCL/MgO nanofiber samples.

The concentration of Mg released from the nanofibers at the time points of 0.5–7 days are shown in Figure 7. The PCL-K/MgO nanofibers had a very high initial burst release of 5.7%, whereas for all other fibers, the initial burst release was only in the range of 0.36%–0.54%. Nanofibers of PCL/MgO ratios of 1:1, 1:1.5 and 1:2 mostly increased slightly in the release of Mg over time. However, for the nanofiber of the PCL-K/MgO ratio of 1:1, the release of Mg decreased over time.

Overall, the nanofiber of the PCL-K/MgO ratio of 1:1 had a greater release of Mg over time compared to the other nanofiber ratios. The cumulative release after seven days for PCL-K/MgO was 15.47%, and for the nanofibers with PCL/MgO ratios of 1:1, 1:1.5 and 1:2, these were 2.28%, 1.76% and 2.01%, respectively. This very high release of Mg^2+^ in PCL-K/MgO is contributed by the hydrophilic nature of keratin, which would cause a higher release of Mg than MgO with PCL alone.

**Figure 8:**
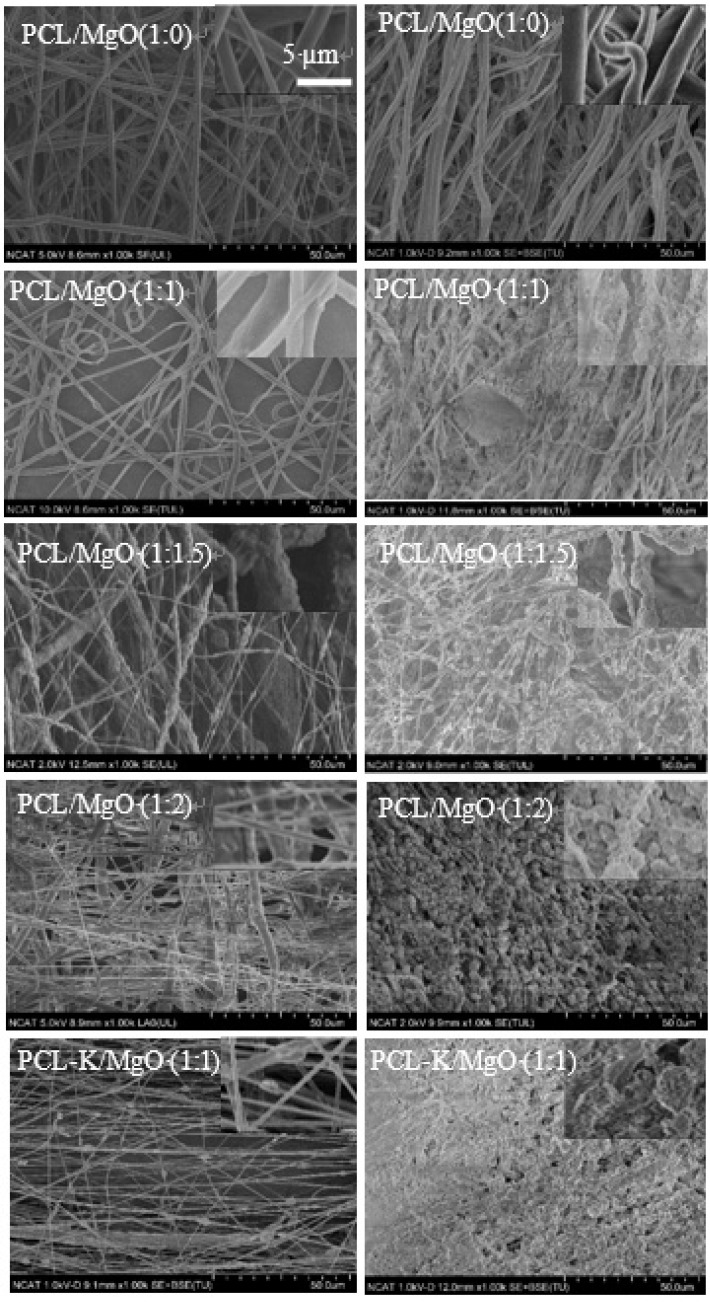
SEM images of *in vitro* degradation of nanofibers. Left images are from Day 0; right images are from Day 7. The scale bar is 50 µm.

The morphology of the PCL/MgO and PCL-K/MgO nanofibers degraded *in vitro* were examined using the SEM. Figure 8 shows the morphological changes of the nanofibers during the degradation study. Representative images of the initial (zero days) and the final (seven days) time periods are displayed.

## 4. Discussion

Dissolving PCL in TFE and keratin powder in DI water both at a concentration of 10% (w/v) was acquired from a previous study [6]. The preparation of a homogeneous polymer solution is one of the most important criteria to obtain uniform polyblend nanofibers of PCL and keratin. In several previous studies, miscible polymer solutions have been achieved with PCL, blended with natural polymers, such as chitosan, collagen, gelatin, silk, *etc*. [6,30,31]. TFE was used in this research, because it has been found to be a good organic solvent in the fabrication of electrospun nanofibers [2]. Through hydrogen bonding, TFE helps in forming stable complexes between PCL and keratin, which then results in the viscosity suitable for electrospinning [6].

In this research, PCL-based composite nanofibers were fabricated by the electrospinning technique. Solutions of PCL/MgO in ratios of 1:0–1:3 were prepared and yielded nanofibers. Overall, the thickness of the pure PCL nanofiber was thicker than the other nanofiber ratios produced, as shown in Figure 1. The distinct result of nanofibers produced from the solution of PCL alone was contributed by solution conductivity and solution viscosity [7]. A decreasing trend in the average diameter of the nanofiber was observed as the ratio of MgO was increased, as shown in Figure 2. Magnesium ions’ presence in the precursor solution enhanced the overall conductivity, which produced smaller diameter nanofiber. Water-soluble keratin extracted from human hair was miscible with PCL and MgO in a PCL-K/MgO ratio of 1:1 and yielded nanofibers. Nanofibers of PCL-K/MgO in a ratio of 1:1 and PCL/MgO in ratios of 1:0–1:2 were further investigated. PCL-K/MgO with a ratio of 1:1 was the composition selected with keratin due to a limited supply of keratin and for the simplicity of this study. The average diameter of the keratin-based nanofiber was higher compared to other nanofibers consisting of MgO. The solutions of PCL-K/MgO with a ratio of 1:1 appeared more viscous compared to the other solutions of PCL/MgO without keratin. This is due to the nature of keratin having recurring integrin-binding domains [19].

In this study, the characteristic FTIR absorption bands at 1720 cm^−1^ and 1625 cm^−1^ confirmed the presence of PCL and keratin, respectively, as shown in Figure 3 [6,29]. PCL/MgO and PCL-K/MgO nanofibers showed the characteristic bands of PCL and keratin. Absorption peaks of MgO are not clearly visible in composite nanofibers, because of their weak intensity compared to PCL and keratin. Peak intensities of keratin are also weak compared to PCL because of its small composition. Keratin proteins give rise to several characteristic absorption bands known as amide I (1600–1700 cm^−1^) and amide II (1480–1580 cm^−1^). The amide I band is mainly associated with the C = O stretching vibration, whereas amide II results from the N–H bending vibration and from the C–N stretching vibration. The characteristic absorption band at amide I at 1625 cm^−1^ was used as a reference peak to confirm the bonding interactions between PCL carbonyl groups and keratin amine groups.

A suitable nanofiber needs to have good mechanical properties for tissue regeneration application. A material modulus close to the target tissue is crucial in order to avoid stress-shielding effect. This allows maintaining the mechanical integrity of the fiber in *in vivo* and *in vitro* applications. The data suggest that PCL nanofibers had the highest Young’s modulus (10.5 MPa) and that PCL-K/MgO nanofibers had the highest ultimate tensile strength (3.2 MPa). The PCL-K/MgO nanofibers had a slightly higher ultimate tensile strength than PCL nanofibers. Young’s modulus and ultimate tensile strength of the PCL-K/MgO nanofibers were significantly higher compared to that of the nanofibers of PCL/MgO with a ratio of 1:1, as shown in Figure 5 and Figure 6. It is expected that a PCL-based composite nanofiber made of natural materials can cause improved mechanical strength compared with nanofibers made of natural or synthetic materials alone [32]. Therefore, a combination of a synthetic polymer (PCL) and a natural polymer (keratin) is the cause of PCL-K/MgO nanofibers having the highest ultimate tensile strength compared to all of the PCL/MgO nanofibers. This is also the reason that PCL-K/MgO nanofibers synthesized in this study had higher mechanical properties compared to PCL/MgO nanofibers with a ratio of 1:1. The increase of strength is due the strong intermolecular hydrogen bonding interactions between PCL carbonyl groups and keratin amine groups. As shown in Figure 5 and Figure 6, the mechanical properties are comparable to those of previously developed PCL-based composite nanofibers for various tissue engineering applications, such as PCL/chitosan [30,33] and PCL/collagen [34].

When PCL/MgO and PCL-K/MgO nanofibers degrade, Mg is released into the degradation medium. The release study of Mg from nanofibers was done with the use of an ICP-OES. PCL-K/MgO nanofiber samples released magnesium significantly more over time compared to all other PCL/MgO nanofiber samples, as shown in Figure 7. However, for the PCL-K/MgO nanofibers, the individual daily release of magnesium decreased over time. PCL is characterized as being hydrophobic, whereas keratin is characterized as being hydrophilic. Due to the hydrophilic nature of the polymer and the presence of hydrolysable amide bonds, keratin shows bulk eroding characteristics, readily allowing permeation of water into the polymer matrix, and degrades throughout the nanofiber [35,36]. Bulk-eroding polymers are often characterized by a burst of drug release during the first few hours of incubation, followed by a slow, diffusion-controlled release [37]. In contrast to keratin, PCL shows surface eroding behavior and degrades very slowly due to the presence of five hydrophobic CH_2_ moieties in its repeating units and a high degree of crystallinity [38]. This is the reason for very low magnesium release in PCL/MgO nanofibers when compared to PCL-K/MgO nanofibers, which shows greater release of magnesium contributed by the high degradation rate of keratin.

The structural integrity for pure PCL nanofiber was unchanged after seven days in PBS medium. However, for other samples, the structural integrity was deformed with the maximum change observed in the PCL-K/MgO sample. This change is supported by the maximum loss of Mg.

The degradation study of Mg demonstrated how PCL/MgO nanofibers and PCL-K/MgO nanofibers would be affected in physiological conditions for a week. At Day 7, the SEM image of the PCL nanofibers did not show any changes, suggesting that the fibers did not degrade. This is consistent with the fact that the polymer PCL can take up to two years to degrade [17]. At Day 7, it can be seen from SEM images for the PCL/MgO nanofibers of ratios of 1:1, 1:1.5 and 1:2 and PCL-K/MgO nanofibers that the fibers retained their surface morphology, as shown in Figure 8. The greater the amount of MgO in the fibers of the PCL/MgO nanofibers, the less intact the fibers were, meaning that the fibers degraded. MgO is a sparingly soluble oxide in water. The degradation of the nanofibers with MgO may not occur in a week, although the fiber morphology changed with a higher composition of MgO. For tissue engineering applications that need a significant amount of time to heal, the degradation time of nanofiber matrix needs to be longer than a week. This rapid degradation was observed when introduced into the PBS solution for over a week, which caused a higher release rate of Mg. In the research discussed by Johnson and Liu (2013), after three days of incubation in PBS solution, magnesium-based samples with oxides on their surface were completely degraded [39]. The degradation of the PCL-K/MgO nanofibers is due to keratin’s hydrophilic nature releasing more Mg into the solution, and then, perhaps, this reaction influences the degradative behavior of the nanofiber.

## 5. Conclusions

PCL/MgO- and PCL-K-MgO-based composite nanofibers were successfully fabricated by using the electrospinning technique. Keratin was effectively extracted from human hair through modification of a published method and was successfully blended with PCL and MgO. The polymer blend solution prepared in the mixture of TFE and DI water resulted in the PCL/MgO and PCL-K/MgO nanofibers with good fiber morphology, suitable mechanical properties and positive magnesium release results. Composite nanofibers were characterized by using SEM, FTIR, mechanical tensile testing and ICP-OES. SEM confirmed that fiber morphology was varied with varying the ratio of MgO and the addition of keratin, too. The characteristic FTIR absorption bands at 1720 cm^−1^ and 1625 cm^−1^ confirmed the presence of PCL and keratin in the fiber. These bands represent the interactions between PCL carbonyl groups and keratin amine groups. The ultimate tensile strength for PCL/MgO nanofiber varied with ratio. As we increased the MgO percentage in the fiber, the tensile strength decreased. Similar results were found when the Young modulus of PCL/MgO nanofiber was compared with PCL nanofiber without MgO. Furthermore, when these mechanical properties of PCL/MgO were compared with PCL-K/MgO nanofibers, it was observed that the keratin-based nanofiber had significantly high values of both the Young modulus and ultimate tensile strength.

The ability to produce nanofibers by combining synthetic and natural polymers with ceramic particulates represents a significant advancement in the development of composite materials with structural and material properties that will support biomedical applications and musculoskeletal tissue engineering.
